# Prevalence and associated factors of meconium aspiration syndrome among neonates admitted to Neonatal Intensive Care Units in Public Hospitals of Harari Region, Eastern Ethiopia

**DOI:** 10.1371/journal.pone.0347861

**Published:** 2026-04-24

**Authors:** Jabir Aman, Bikila Balis, Naol Oda, Dawit Tamiru, Tadesse Gure Eticha, Dawit Firdisa, Aboma Motuma

**Affiliations:** 1 Hiwot Fana Comprehensive Specialized Hospital, College of Health and Medical Science, Haramaya University, Harar, Ethiopia; 2 School of Midwifery, College of Health and Medical Sciences, Haramaya University, Harar, Ethiopia; 3 School of Medicine, College of Health and Medical Sciences, Haramaya University, Harar, Ethiopia; 4 School of Public Health, College of Health and Medical Sciences, Haramaya University, Harar, Ethiopia; 5 School of Nursing and Midwifery, College of Health and Medical Sciences, Haramaya University, Harar, Ethiopia; Haramaya University Faculty of Health Sciences: Haramaya University College of Health and Medical Sciences, ETHIOPIA

## Abstract

**Background:**

Meconium aspiration syndrome is a life-threatening respiratory disease affecting around 5% of neonates worldwide. Although several studies have been conducted in developed countries, data on meconium aspiration syndrome and its associated factors remain limited in low-resource settings, including Ethiopia. Therefore, this study aimed to determine the meconium aspiration syndrome and associated factors among neonates admitted to the neonatal intensive care unit at public hospitals in Harari region, Eastern Ethiopia.

**Method:**

A retrospective hospital-based cross-sectional study design was conducted among all neonates admitted from January 1 to December 30, 2023 and data were extracted from patient charts during April 1–30, 2025. A simple random sampling technique was employed to select 417 charts of neonates admitted to the neonatal intensive care unit. The data were collected by a data extraction checklist via Kobo Toolbox. Descriptive statistics and binary logistic regression were used in SPSS version 25 (IBM Corp., Armonk, NY, USA) for the analysis. Adjusted odds ratios with 95% confidence intervals were used to declare statistical significance at a p-value ≤ 0.05.

**Results:**

The prevalence of meconium aspiration syndrome among neonates admitted to the neonatal intensive care unit was 24.2% [95% CI, 20.2–28.6]. Factors significantly associated with meconium aspiration syndrome were post-term gestation [AOR = 9.05, 95% CI 2.38–34.41], antepartum hemorrhage [AOR = 3.34, 95% CI 1.31–8.60], prolonged labor [AOR = 3.06, 95% CI 1.27–7.36], premature rupture of membranes [AOR = 3.65, 95% CI 1.28–10.45], low Apgar scores at 5th minute [AOR = 11.27, 95% CI 3.44–36.92] and intrapartum thick meconium passage [AOR = 5.98, 95% CI 2.6–13.6].

**Conclusions and recommendations:**

These findings indicate a high prevalence of meconium aspiration syndrome, and to reduce its impact, targeted clinical interventions should be implemented. Pregnancies reaching 42 weeks of gestation, prolonged labor, and high-risk conditions such as antepartum hemorrhage, premature rupture of membranes, or the presence of thick meconium are important factors to consider. Careful monitoring and appropriate management may be warranted in these cases.

## Introduction

Meconium-stained amniotic fluid (MSAF) is considered an alarming sign of possible fetal distress linked with an adverse perinatal outcome [1]. Passage of meconium in newborns is a normal developmental event, typically occurring within the first 24–48 hours after birth [2].

Globally, meconium aspiration syndrome (MAS) is a life-threatening respiratory disease occurring when neonates aspirate meconium-stained amniotic fluid near or around birth with varying severity [3]. An average of 8% to 25% of live birth neonates with meconium-stained amniotic fluid (MSAF) have delivery complications and from these around 5% of them develop MAS with a 50% chance of needing mechanical ventilation [4]. Each year, 2.6 million neonates die worldwide, with three-fourths of these deaths occurring in the first week [5,6]. The issue of neonatal mortality and morbidity is a priority of the sustainable development goals (SDG) with the goal of lowering neonatal fatalities to less than 12 deaths per 1000 live births by 2030 [7]. Around 11 million lives would be saved if all nations in the world met the SDGs’ goal of decreasing under-five fatalities, particularly newborn deaths, with more than half of those saved in Sub-Saharan Africa [8,9]. Ethiopia has one of the worst rates of newborn mortality in the world; MAS accounts for a significant portion of these deaths, with the early neonatal period accounting for approximately 80% of all neonatal deaths [10,11].

The exact causes of MAS remain unclear, but significant risk factors include maternal obstetric conditions, neonatal characteristics, and demographic factors [12]. Factors associated with MAS include primiparous, preeclampsia [13], maternal age [2], prolonged labor, premature rupture of membranes, cesarean delivery, advanced gestation [14], and indicators of neonatal distress such as low Appearance, pulse, grimace, activity, respiration (Apgar) scores and abnormal fetal heartbeats [15]. In order to minimize the incidence of MAS, intrapartum care and coordination of care between the obstetricians and neonatal teams are crucial, whereas Ethiopian national recommendations advise appropriate prenatal care [16].

Despite significant progress in reducing maternal and child mortality through various initiatives over the past two to three decades, newborn mortality increased to 33 deaths per 1,000 live births in 2019, rising from 29 per 1,000 in 2016 [17,18]. Ethiopia aims to decrease neonatal mortality to 11 per 1,000 by 2019–2020, but current figures indicate a deterioration, with 60% of infants facing higher risks from preventable factors [19]. Although several studies have been conducted in developed countries, evidence on the magnitude and risk factors of meconium aspiration syndrome remains limited in developing countries like Ethiopia, particularly in the study area [12]. In addition, most cases of MAS require close observation and treatment, making neonatal intensive care units (NICUs) an essential platform for identifying MAS cases and determining associated risk factors [20]. However, little is known about MAS cases among neonates who are admitted to NICUs in public hospitals located in the Harari region. Therefore, this study aimed to determine the prevalence of meconium aspiration syndrome and its predictors in neonates admitted to the neonatal intensive care unit at public hospitals in Harari region, Eastern Ethiopia.

## Methods and materials

### Study setting, design, and period

A retrospective hospital-based cross-sectional study was employed in two public hospitals of Harari region, Eastern Ethiopia, namely Hiwot Fana Comprehensive Specialized Hospital (HFCSH) and Jugal General Hospital. The population under study comprised all neonates admitted from January 1 to December 30, 2023 and data were extracted from patient charts during April 1–30, 2025. The Harari region is located 526 km away from the capital city, Addis Ababa. Hiwot Fana Comprehensive Specialized University Hospital was affiliated with Haramaya University College of Health and Medical Sciences. It serves more than 1800 neonatal admissions annually. Jugal General Hospital is one of the public hospitals found in Harari region, which was established as the first public hospital in Ethiopia in 1956 as a memorial for Ras Mekonen. Currently, it is serving as a regional hospital with a catchment population of 500,000.

### Population and sampling

All neonates admitted to the neonatal intensive care unit at public hospitals in Harari region were the source population, whereas neonates admitted to the neonatal intensive care unit at public hospitals in Harari region from January 1 to December 30, 2023, are the study populations. All neonates who were admitted to Neonatal Intensive Care Unit (NICU) from January 1 to December 30, 2023, and whose medical charts contained at least patient identification, obstetric history, neonatal birth history, and medical diagnosis of the neonate were included, whereas neonates who had no complete information, neonates with lost cards, born before 28 completed weeks of gestation, and home delivery were excluded.

The required sample size was calculated by using both the single population proportion and double population proportion formulas to address the two objectives of the study. The sample size for a single population proportion sample size (n) was calculated by taking the previous prevalence of meconium aspiration syndromes in Northwest Ethiopia, 55.6% [21], marginal error (d = 5%), and 95% confidence interval (Zα/2 = 1.96). $n=\;\frac{{\frac{{(Z}^{\alpha }}{\text{2})}}^{\text{2}}\;P(\text{1}-P)\;}{{\text{d}}^{\text{2}}}$, n = 379. By adding 10% non-response rate, the final sample size was 417. Double population proportion formula was used to determine the sample size for associated factors from previous studies by taking three associated variables, namely, obstructed labor [12], Premature rupture of membrane (PROM) [12], and post-term pregnancy [21], by using EPI Info version 7.2.6 menu Stat-calc, by considering the following assumptions: confidence level 95%, power 80%, and exposed to an unexposed ratio of 1:1. Finally, the required sample size for this study was decided by taking the maximum sample size from the two calculations. Therefore, the final sample size (n) was taken from a single population proportion, which is 417.

Regarding the selection of hospitals, two public hospitals that were found in the Harari region were included. To get the study participants, first, the total number of neonates who were admitted from January 1 to December 30, 2023, was obtained from the neonatal admission registry logbook of each hospital. Based on the annual reports, the number of neonatal admissions in Jugal General Hospital was 320, and in the case of HFCSH, the annual report numbers of neonatal admission were 1560, with a total of 1880. The sampling population was selected from each hospital by proportional allocation. Then the study unit was selected by a simple random sampling technique using a lottery method after getting the sampling frame from the neonatal admission registry chart lists.

### Operational definition

**MAS:** Meconium Aspiration Syndrome is diagnosed by a pediatrician based on having all three clinical parameters: 1) being born through MSAF, 2) having an abnormal chest x-ray, and 3) having respiratory distress [13].

**Preterm gestation:** gestational age < 37 completed weeks of pregnancy [22].

**Post-term gestation:** gestational age ≥ 42 weeks of pregnancy [22].

**Small for gestational age:** It is related to birth weight and gestational age if the birth weight is less than the 10th percentile, as diagnosed by a pediatrician/residents [23].

**Thick meconium:** heavy staining occurs when there is reduced amniotic fluid and a large amount of meconium, making the staining quite thick, with a “pea soup” consistency [15].

**Thin meconium:** amniotic fluid diluted with meconium, with a large to moderate amount of amniotic fluid. Just meconium-stained amniotic fluid [15,24].

**Prolonged labor**: it was calculated by adding the number of hours the woman was laboring before admission, which was taken from her history, and the time she stayed in the hospital until delivery. If labor duration lasts more than 12 hours [12,15].

**Non-reassuring fetal heart rate pattern (NRFHRP):** defined as fetal heart rate of <120 or >160 beats per minute that stays for more than 15 minutes [21].

### Data collection and quality control

The structured data extraction checklist was developed after reviewing relevant literatures to the problem under study [2,12,14,24,25]. The structured data extraction checklist has three sections including; socio demographic factors (maternal age**,** sex of neonate, Age of neonate), obstetrics related factors (Gravidity, Parity, Gestational age, Antenatal care (ANC) follow up, Anemia, Pre-eclampsia, Antepartum hemorrhage (APH), Gestational Diabetes mellitus (GDM), Oligohydramnios, Induction of labor, Infection, Obstructed labor, PROM and Duration of labor), and Neonatal factors (Fetal presentation, Birth weight, Fetal distress, Intra-uterine meconium release, 1^st^ mint Apgar score, 5^th^ mint Apgar score). Two trained BSc midwives collected data from patients’ charts by using a data extraction checklist, using Kobo Toolbox. Neonatal charts and registration books were retrospectively reviewed from April 1–30, 2025 for neonates admitted between January 1 and December 31, 2023. The principal investigator, the supervisor, and the data collectors discussed and resolved the problems that arose during data collection. Data were collected by trained data collectors after training was given on the general purpose, data collection methods, and data handling techniques. Charts with missing information on one or more of the following key variables: place of delivery, maternal obstetric history, or neonatal birth history were considered incomplete and excluded from the study [23].

The pre-test was conducted on 5% of the sample size in Haramaya General Hospital, which is outside the study area. Data collected were checked daily for completeness and consistency by the principal investigators and one MSc midwife supervisor. In addition, data collectors and supervisors were trained for two days on how to collect data from neonatal charts and check for the accuracy, consistency, and completeness of all variables.

### Data processing and analysis

The collected data were cleaned and coded after exported to SPSS version 25 (IBM Corp., Armonk, NY, USA) for analysis. Descriptive analyses such as percentages, frequency distribution, and measures of central tendency like mean and standard deviation were done. Then bi-variable analysis between dependent and independent variables was performed. Finally, those variables showing association at a p-value less than 0.25 were considered in a multivariable logistic regression analysis to control possible confounding and to identify independent predictor variables of meconium aspiration syndrome [23]. The multicollinearity test was checked by using the variance inflation factor (VIF). The mean VIF was 1.915, and all variables had VIF values ranging from 1.12 to 2.34. The goodness of model fit was checked by the Hosmer-Lemeshow model fit-test (P-value = 0.907). The logistic regression model demonstrated high predictive utility. The final model correctly classified 88% of cases overall, with a sensitivity of 91.7% and a specificity of 82.2% (at a 0.50 cut-off). The discriminatory power of the model was evaluated using the Receiver Operating Characteristic curve, and the area under the curve (AUC) was 0.929, indicating good discrimination. Interaction between independent variables was assessed, and no significant interaction effects were identified. To declare statistical significance adjusted odds ratio with 95% confidence intervals (CI), and a p-value < 0.05 was used.

### Ethics approval and informed consent

The study was conducted according to the Declaration of Helsinki. Ethical clearance was obtained from the Institutional Health Research Ethics Review Committee (IHRERC) of the College of Health and Medical Sciences, Haramaya University, with reference number IHRERC/058/2025. Informed, voluntary, written, and signed consent was obtained from the head of hospitals. After obtaining consent from each hospital administrator, the data collection commenced. Informed consent from patients was waived due to the retrospective nature of the chart review, and all data were anonymized and de-identified prior to analysis. All information obtained from the participants’ charts was kept confidential.

## Results

### Socio-demographic profile of the mothers and neonates

This study included 417 participants with a 100% response rate. The finding of this study revealed that mothers whose neonates were admitted to the NICU had a mean age of 25 years with a standard deviation of ± 4.7 (range: 16–39 years). The majority of them were between the ages of 20 and 24 years, comprising 146 (35%) of the total, followed by those aged 25–29 years, 136 (32.6%). Regarding place of residence, most of the mothers, 266 (63.8%), came from rural areas, while 151 (36.2%) were from urban settings. Regarding neonates, the majority, 357 (85.6%), were admitted to the NICU within the first seven days of life; 60 (14.4%) were between 7 and 28 days old. In terms of sex, female neonates slightly outnumbered males, accounting for 233 (55.9%) compared to 184 (44.1%) male neonates (Table 1).

**Table 1:** The socio-demographic characteristics of mothers and neonates admitted to NICU at public hospitals in Harari region from January 1 to December 30, 2023 (n = 417).

Variables	Categories	Frequency	Percent (%)
Maternal age (year)	15-19	41	9.8
	20-24	146	35.0
	25-29	136	32.6
	30-34	84	20.1
	≥35	10	2.4
Residence	Urban	151	36.2
	Rural	266	63.8
Region of participants	Harari	123	29.5
	Oromia	276	66.2
	Others	18	4.3
Age of Neonate	<7 days	357	85.6
	≥7 days	60	14.4
Sex of Neonate	Male	184	44.1
	Female	233	55.9

**Others**: Somali region and Dire Dawa City.

**Statistical tests used**: Descriptive statistics (frequency and percentage).

### Characteristics of neonates admitted to NICU

The majority of neonates admitted to the NICU during the study period, 308 (73.8%), had normal birth weights ranging from 2500 to 3999 grams, while 66 (15.8%) were classified as small for gestational age (SGA). In terms of immediate postnatal health, 238 (57.1%) of the neonates had an Apgar score of 7 or less at the first minute. However, at the fifth minute, 228 (54.6%) achieved an Apgar score of 7 or higher, showing some recovery. Most neonates were delivered in vertex presentation, 358 (85.9%). Intrapartum meconium release occurred in 117 (28.1%) of deliveries, with 53 (45.3%) having thin (grade 1) meconium and 64 (54.7%) having thick (grades 2 or 3) meconium (Table 2).

**Table 2:** Characteristics of Neonates Admitted to NICU at public hospitals in Harari region from January 1 to December 30, 2023 (n = 417).

Variables	Categories	Frequency	Percent (%)
Newborn birth weight in gram	<2500	80	19.2
	2500-3999	308	73.8
	≥4000	29	7
SGA	Yes	66	15.8
	No	351	84.2
Apgar score at-1st minute	≥7	179	42.8
	<7	238	57.2
Apgar score at-5th minute	≥7	228	54.6
	<7	189	45.4
Fetal Presentation	Vertex	358	85.9
	Non-vertex	59	14.1
Birth Asphyxia	Yes	118	28.3
	No	299	71.7
Early neonatal sepsis	Yes	263	63.1
	No	154	36.9
Respiratory distress syndrome	Yes	52	12.5
	No	365	87.5
NRFHRP	Yes	100	24.0
	No	317	76.0
Intrauterine meconium release	Yes	117	28.1
	No	300	71.9
Grades of meconium (n = 117)			
Grade of meconium	Thin (G1)	53	45.3
	Thick (G2 and 3)	64	54.7
Neonatal complication secondary to MAS (n = 208)			
Neonatal complication secondary to MAS	Jaundice	2	1
	Birth asphyxia	64	30.8
	Hypoglycemia	10	4.8
	Hypothermia	34	16.3
	Sepsis	98	47.1
Presence of congenital anomaly	Yes	24	5.8
	No	393	94.2
Newborn Resuscitation done	Yes	114	27.3
	No	303	72.7
Oxygen supporting done	Yes	89	21.3
	No	328	78.7

**Note:** Percentages for meconium grades are based on neonates with meconium-stained amniotic fluid (n = 117); percentages for neonatal complications are based on neonates presenting with at least one complication secondary to MAS (n = 208); all other variables use the total sample (N = 417).

Abbreviations; SGA: small for gestational age, MAS: meconium aspiration syndromes, NRFHRP: non-reassuring fetal heart rate pattern, Apgar: Appearance, Pulse, Grimace, Activity, Respiration, G1: grade 1, G2: grade 2, and G3: grade 3.

**Statistical tests used:** Descriptive statistics (frequency and percentage).

### Obstetric-related factors

The majority of mothers whose neonates were admitted to the NICU were multiparous, accounting for 271 (65.0%). In terms of gestational age, most deliveries were at term: 279 (66.9%) deliveries. Regarding ANC, the majority of mothers, 336 (80.6%), had ANC follow-up. Preeclampsia was the most common pregnancy complication, 127 (30.5%). Gestational diabetes mellitus was noted in 22 (5.3%). Additionally, regarding labor duration, 158 (37.8%) of mothers experienced prolonged labor lasting 12 hours or more. Moreover, PROM was documented in 122 (29.3%), and obstructed labor was observed in 77 (18.5%) of mothers (Table 3).

**Table 3:** Obstetrics factors of mothers whose neonates admitted to NICU at public hospitals in Harari region from January 1 to December 30, 2023 (n = 417).

Variables	Category	Frequency	Percent (%)
Parity	Primipara	146	35.0
	Multipara	271	65.0
Gestational age in week	Preterm	79	19
	Term	279	66.9
	Post term	59	14.1
Antenatal care follow-up	Yes	336	80.6
	No	81	19.4
Number of ANC follow-up	<4 visit	111	33
	≥4 visit	225	67
Preeclampsia	Yes	127	30.5
	No	290	69.5
Antepartum hemorrhage	Yes	63	15.2
	No	354	84.8
Gestational diabetes mellitus	Yes	22	5.3
	No	395	94.7
Oligohydramnios	Yes	64	15.3
	No	353	84.7
Induction of labor	Yes	119	28.5
	No	298	71.5
Infection	Yes	85	20.4
	No	332	79.6
Mode of delivery	Spontaneous	224	53.8
	Instrumental	80	19.2
	Cesarean	113	27.0
Duration of labor	≥12hr	158	37.8
	<12hr	259	62.2
Place of delivery	Public hospital	304	73.0
	Health center	87	20.8
	Private facility	26	6.2
PROM	Yes	122	29.3
	No	295	70.7
Obstructed labor	Yes	77	18.5
	No	340	81.5
Multiple pregnancy	Yes	20	4.8
	No	397	95.2
Anemia	Yes	183	43.9
	No	234	56.1

**Abbreviations;** ANC: Antenatal care, PROM: premature rupture of membrane.

**Statistical tests used**: Descriptive statistics (frequency and percentage).

### Magnitude of meconium aspiration syndrome

This study’s findings showed that the prevalence of meconium aspiration syndrome among neonates admitted to NICU was 101(24.2%) [95% CI, 20.2, 28.6].

### Factors associated with meconium aspiration syndrome (MAS)

In multivariable analysis, post-term gestation, antepartum hemorrhage, prolonged labor of 12 hours or more, premature rupture of membranes, low Apgar scores at the 5th minute, and intrapartum thick meconium passage remained significantly associated with meconium aspiration syndromes.

The odds of developing MAS is 9 times higher among post-term neonates compared to those born preterm [AOR = 9.05, 95% CI 2.38–34.41]. Similarly, the presence of antepartum hemorrhage was associated with a more than threefold increase in the likelihood of MAS [AOR = 3.34, 95% CI 1.31–8.60]. Prolonged labor of 12 hours or more significantly increased the odds of MAS, with affected neonates being three times more likely to develop the condition than those born following <12 hours labor durations [AOR = 3.06, 95% CI 1.27–7.36]. Likewise, neonates born after PROM had a 3.65 times higher likelihood of developing MAS [AOR = 3.65, 95% CI 1.28–10.45] than their counterparts. A particularly strong association was observed between MAS and low Apgar scores at the 5th minute. Neonates with scores below 7 were over eleven times more likely to develop MAS than those with higher scores [AOR = 11.27, 95% CI 3.44–26.92]. Intrapartum thick meconium passage was also significantly associated with MAS, with a 6 times higher risk compared to those with a thin meconium passage [AOR = 5.98, 95% CI 2.6–13.6] (Table 4).

**Table 4:** Bivariable and multivariable logistic regression analysis of meconium aspiration syndrome and associated factors among neonates admitted to NICU at public hospitals in Harari region from January 1 to December 30, 2023 (n = 417).

Variables	Category	MAS Yes (%)	MAS No (%)	COR (95% CI)	AOR (95%CI)
Parity	Primipara	51 (12.2%)	95 (22.8%)	2.37 (1.50-3.75)	1.78 (0.94-3.35)
	Multipara	50 (12%)	221 (53%)	1	1
Gestational Age	Post term	34 (8.2%)	25 (5.9%)	6.91 (3.14-15.18)	9.05 (2.38-24.41)**
	Term	54 (12.9%)	225 (54%)	1.22 (0.63-2.37)	1.39 (0.47-4.06)
	Pre-term	13 (3%)	66 (16%)	1	1
Preeclampsia	Yes	62 (14.8%)	65 (15.6%)	6.14 (3.78-9.97)	1.23 (0.56-2.75)
	No	39 (9.4%)	251 (60.2%)	1	1
Oligohydramnios	Yes	37 (8.9%)	27 (6.5%)	6.19 (3.52-10.89)	0.95 (0.37-2.47)
	No	64 (15.3%)	289 (69.3%)	1	1
Antepartum hemorrhage	Yes	33 (8%)	30 (7.2%)	4.63 (2.64-8.11)	3.34 (1.31-8.60)*
	No	68 (16.3%)	286 (68.5%)	1	1
Duration of labor	≥12hr	82 (19.6%)	76 (18.2%)	13.63 (7.77-23.9)	3.06 (1.27-7.36)*
	<12hr	19 (4.6%)	240 (57.6%)	1	1
Obstructed labor	Yes	45 (10.8%)	32 (7.7%)	7.13 (4.17-12.19)	1.58 (0.18-1.72)
	No	56 (13.4%)	284 (68.1%)	1	1
Premature rupture of membrane	Yes	73 (17.5%)	49 (11.8%)	14.2 (8.35-24.17)	3.65 (1.28-10.45)*
	No	28 (6.7%)	267 (64.0%)	1	1
APGAR score at 5^th^ minute	<7	82 (19.7%)	107 (25.7%)	8.43 (4.86-14.62)	11.27 (3.44-26.92)**
	≥7	19 (4.5%)	209 (50.1%)	1	1
Grade of intrauterine meconium released	Thick(G2,3)	51 (43.6%)	13 (11.1%)	5.98 (2.6-13.6)	3.16 (1.25-7.96)*
	Thin(G1)	21 (17.9%)	32 (27.4%)	1	1

**Note:** * p ≤ 0.05, ** p ≤ 0.001. COR: Crude Odds Ratio, AOR: Adjusted Odds Ratio, CI: Confidence Interval, MAS: Meconium Aspiration Syndrome.

**Statistical tests used:** Bivariate and multivariable binary logistic regression.

## Discussion

MAS is a challenging problem and one of the most common causes of respiratory distress among neonates, which is the main reason for NICU admission. The prevalence of meconium aspiration syndrome found in this study was 24.2% [95% CI: 20.2–28.6]. Being post-term gestation, antepartum hemorrhage, prolonged labor of 12 hours or more, premature rupture of membranes, low Apgar scores at 5th minute, and intrapartum thick meconium passage were factors significantly associated with meconium aspiration syndrome.

The prevalence observed in this study is comparable with findings reported from other studies conducted in Ethiopia. For instance, the study conducted at Jimma University Specialized Hospital, reported a prevalence of 19.9% [14], and at Wolkite University Specialized Hospital (28.7%) [24], and it is consistent with the study done in the Amhara region at northwest comprehensive specialized hospitals which resulted in (23.6%) [23].

However, the finding of this study was higher than the study conducted in Australia and New Zealand, which revealed rates of MAS as low as (3.5%) [26] and in Pakistan (14.9%) [27], in India (8.5%) [28]. Such a difference may be explained by the varying healthcare systems, study groups, and perinatal care practices. High-income countries have a low incidence of MAS due to better antenatal monitoring, continuous fetal monitoring during labor, and prompt medical interventions. Evidence revealed that the implementation of evidence-based obstetric and neonatal care practices in developed countries has reduced the incidence of MAS [29]. In contrast, the increased prevalence in low- and middle-income countries may be due to a lack of healthcare resources, delayed access to skilled obstetric care, and referral of complicated cases to tertiary centers, thereby increasing the likelihood of MAS [20]. Furthermore, some studies conducted in developed countries are population-based, whereas the current study is NICU-based, including more severe or complicated pregnancies, which may explain the higher prevalence of MAS [30].

On the other hand, the magnitude of MAS in this study was lower than that in the study conducted in Turkey (60.3%) [31], in India (34%) [32], and in Southern Ethiopia at Wachemo University (30.6%) [12]. The larger figure observed in the Turkish and Indian studies might be because of variations in the study denominator, as MAS was calculated among neonates with MSAF, while in the present study, all NICU admissions were included, making it a smaller proportion. Also, small variations in sample size, study design, data collection methods, and clinical practices might have contributed to a higher prevalence observed in the Wachemo study compared to the present study.

In this study, post‑term gestation exhibited higher odds of MAS compared to preterm gestation. The findings from this study were supported by previous studies conducted in China [33], in Nepal [34], in France [35], in Turkey [31], and in Ethiopia [23,36]. This might be due to fetal maturation, placenta insufficiency, and resultant fetal hypoxia and acidosis may be the cause of this. These factors might relax the anal sphincter, allowing meconium passage and aspiration [23,34]. Therefore, once the fetus reaches 42 weeks and beyond, the gastrointestinal system will mature, and the fetus may suffer meconium aspiration syndrome. So, the neonate was negatively impacted by being post-term. This indicates that preventing post-term pregnancy and doing elective delivery may reduce the risk of MAS for clinical implications.

This study also found that neonates born to mothers who had antepartum hemorrhage were about more than 3 times more likely to develop meconium aspiration syndrome compared to those born to mothers who did not have an antepartum hemorrhage. This is in line with the findings from studies done in Southern Ethiopia [12], and also supported by a study done at Nigist Eleni Mohammed Memorial Comprehensive Specialized Hospital, South Ethiopia [37]. A possible cause could be utero-placental ischemia, which may cause fetal distress and is strongly linked to intrapartum meconium discharge [12].

The odds of MAS increased about 3 times more in neonates whose mother’s duration of labor was greater than or equal to 12hr to that whose mother’s duration of labor was less than 12 hours. This finding is in line with the study done at Northwest Ethiopia comprehensive specialized hospitals [23], in South Ethiopia [24], and also this finding is supported by a previous study done at Nigist Eleni Mohammed Memorial Comprehensive Specialized Hospital, South Ethiopia [37]. The most likely explanation for this could be that a prolonged labor may increase the likelihood of fetal distress, due to prolonged uterine contractions and reduced oxygen supply to the fetus, which may stimulate meconium passage before labor [37]. Another studies explained that prolonged labor may cause fetal distress and hormonal changes (e.g., increased cortisol), which increase colonic motility and result in meconium passage [20,38]. It implies that to prevent such risks, following proper obstetric care, such as advising the woman to arrive early during labor and making early decisions for intervention, accessing the emergency obstetric care functionality to the level of a health center, like emergency cesarean section, can lower the incidence of prolonged labor duration and accomplish sustainable development goals.

Furthermore, this study showed that neonates whose mothers had PROM were more prone to have MAS as compared to those who didn’t have PROM. This study was consistent with the study conducted in southern Ethiopia [12], in Nigist Eleni Mohammed Memorial Hospital, South Ethiopia [37], and in northwest Ethiopia’s comprehensive specialized hospitals, Northwest Ethiopia [23]. This might be attributed to PROM, which might cause the fetus to receive insufficient fluid and oxygen, cord compression, and prolapse, which causes gasping and fetal distress. Fetal hypoxia can occur during labor and delivery or in utero, which might result in aspiration of the MSAF. Furthermore, when the membrane is ruptured before labor, the fetus may have a high chance of swallowing the meconium-stained amniotic fluid. This increases the likelihood of deliveries through inducement of labor, which may increase fetal suffering due to tetanic uterine contraction following oxytocin administration.

This study found that intrapartum thick meconium passage was significantly associated with meconium aspiration syndrome. Several studies from India [28,39] and in South Ethiopia [24] also reported the same findings. Because of this, a thick meconium may potentially narrow or obstruct the airway. This might be explained by the fact that aspiration of thick meconium may potentially narrow or obstruct the airway.

The findings of this study showed that neonates having low Apgar scores at five minutes were significantly associated with meconium aspiration syndrome. This finding was supported by a study done in India [39,40], in Southern Ethiopia and south-west Ethiopia [14,24]. This might be from the outcome of delayed early neonatal resuscitation practices, which lead to a low fifth-minute Apgar score, or the relevance of the amount of meconium in the amniotic fluid as a symptom of intrauterine fetal impairment or perinatal hypoxia.

### Strength of the study

Since it uses secondary data, it is cost-effective and time-saving. In addition to this, hospital records may have data from many patients over a long period of time, improving statistical power and representativeness.

### Limitation of the study

Participants or variables may be incorrectly categorized due to errors in documentation. Generalizability may be limited, since the study relies on hospital records, which might be subject to biases in documentation or under-reporting of meconium aspiration syndrome. Additionally, cross-sectional nature of the study limits the ability to establish a clear temporal relationship between exposure variables and the outcome.

## Conclusion

The prevalence of meconium aspiration syndrome in this study differs from the 5% estimate reported by World Health Organization [41]. Post-term pregnancy, antepartum hemorrhage, prolonged labor, premature rupture of membranes, low 5th-minute Apgar score, and thick meconium-stained amniotic fluid were significant predictors. These findings highlight the need of addressing these risk factors through targeted interventions, improving maternal and neonatal healthcare access, early detection, and appropriate intervention to decrease MAS. Additionally, future research using primary data is recommended to assess some predictors variables missed in secondary data, such as maternal occupation, educational status, maternal body mass index (BMI), smoking, khat chewing, alcohol consumption, income, etc.

## Supporting information

S1 File Minimal data set. (SAV)
